# The development of honey bee colonies assessed using a new semi-automated brood counting method: CombCount

**DOI:** 10.1371/journal.pone.0205816

**Published:** 2018-10-16

**Authors:** Théotime Colin, Jake Bruce, William G. Meikle, Andrew B. Barron

**Affiliations:** 1 Department of Biological Sciences, Macquarie University, Sydney, New South Wales, Australia; 2 School of Electrical Engineering and Computer Science, Queensland University of Technology, Brisbane, Australia; 3 Carl Hayden Bee Research Center, USDA-ARS, Tucson, Arizona, United States of America; University of North Carolina at Greensboro, UNITED STATES

## Abstract

Precise, objective data on brood and honey levels in honey bee colonies can be obtained through the analysis of hive frame photographs. However, accurate analysis of all the frame photographs from medium- to large-scale experiments is time-consuming. This limits the number of hives than can be practically included in honeybee studies. Faster estimation methods exist but they significantly decrease precision and their use requires a larger sample size to maintain statistical power. To resolve this issue, we created ‘CombCount’ a python program that automatically detects uncapped cells to speed up measurements of capped brood and capped honey on photos of frames. CombCount does not require programming skills, it was designed to facilitate colony-level research in honeybees and to provide a fast, free, and accurate alternative to older methods based on visual estimations. Six observers measured the same photos of thirty different frames both with CombCount and by manually outlining the entire capped areas with ImageJ. The results obtained were highly similar between both the observers and the two methods, but measurements with CombCount were 3.2 times faster than with ImageJ (4 and 13 min per side of the frame, respectively) and all observers were faster when using CombCount rather than ImageJ. CombCount was used to measure the proportions of capped brood and capped honey on each frame of 16 hives over a year as they developed from packages to full-size colonies over about 60 days. Our data describe the formation of brood and honey stores during the establishment of a new colony.

## Introduction

The number and intensity of stressors of honeybees has increased considerably in the last decades and may continue to increase in the near future [1]. It is therefore important to understand the process of development of commercial honey bee colonies, and how that development is impacted by different stressors [2,3]. When assessing colony development and productivity, key parameters of interest include the number of bees, the amount of brood and the mass of honey stored in the hive. Pollen is also a crucial nutrient for honey bees, but pollen stores have a high turnover rate compared to honey, with most pollen being consumed within 96 h of its collection [4]. Pollen surface area may thus not reflect pollen influx and use, and is less often estimated.

To date, studies that have focused on brood and honey production have either estimated or manually measured the amount of brood or honey in the hive [5–15]. Different methods present different problems. The amount of brood can be estimated using the Liebefelder method [16] which is a visual estimation of the surface of the brood of every frame. The variation within datasets and between observers is likely to be higher with this method than with direct counts. It has been stressed that the Liebefelder method requires intensive training before a user can give reliable estimates without placing a grid in front of the frames [17]. Fresnaye and Lensky [18] compared three methods: a visual estimation similar to the Liebefelder method, a measure of the small and large axis of the brood patch to estimate the area of the ellipsis containing the brood, and the use of a planimeter and a grid similar to the one recommended for inexperienced users in the Liebefelder method. They found on average 23.3% of error when estimating the surface of the brood areas visually with up to 78.5% of error per frame, 13.3% of error when calculating the area of an ellipsis and 14.8% of error when measuring the brood with a planimeter and a grid similar to the one recommended for the Liebefelder method. These high error rates can only decrease the power of statistical analyses, forcing researchers to increase their sample size.

The use of photography to measure brood has been considered since 1924 [18]. Frame photographs can be evaluated with precision and are themselves permanent records of the data, but the technical constraints of film photography made this impractical for a large number of hives. With the development of digital photography, methods have been developed to measure the area covered by capped brood cells on photos either with Photoshop [19], ArcView [20] or ImageJ [13,21,22], although obtaining fast and accurate results is often challenging, especially when the cells are scattered across a frame rather than clustered.

Current methods to accurately quantify capped brood and capped honey are slow, and this may limit sample size [11] in colony-level studies in honeybees. Studies involving mature colonies have often been conducted on species that live in small colonies of a few hundred individuals such as bumblebees [14,23] or certain ants [24,25] where individuals can easily be counted, but they remain uncommon for honeybees because the scale of a bee hive poses real problems for experimental assessment or manipulation [3]. Typically, researchers reduce the size of colonies for use in their experiments on honey bee diseases and stressors [26–31], or focus on individual or small groups of bees *in vitro* [32,33]. However, application of results from studies done with small colonies or groups of bees in controlled or laboratory settings to full-size bee colonies in the field may be more complex than simply scaling up due to the increased number and kinds of interactions among members [34]. These concerns have been recently discussed [3,31]. Decreasing the time and practical costs of colony-level studies is consequently a better long-term strategy for research on honeybee health than accepting the limitations of studies on individual honeybees.

Here CombCount (an open-source program for Python) was used to facilitate measurement of capped brood and capped honey both in the field when photographing frames and in the lab when analysing them. CombCount can be used on Windows, Macintosh and Linux. CombCount detects uncapped cells but does not automatically differentiate capped honey from capped brood. After capped brood and capped honey areas have been identified visually by the user, wide areas can be quickly drawn around the capped brood and capped honey to measure capped brood and capped honey areas. CombCount can also be configured to fit a wide range of lighting conditions. The time saved, and the accuracy of CombCount compared to ImageJ were measured across six observers on a sample of 30 frames.

CombCount was then used to measure capped brood and capped honey stores to track development of new honey bee colonies from commercial packages of honey bees. A package of bees contains 2kg of workers and a young mated queen, similar to a swarming group. These are sold commercially and commonly used by bee keepers to establish new commercial colonies. We analysed 1664 photos of frames taken from 16 hives across 8 hive evaluations. Empirical and theoretical studies on the organization of brood, pollen and nectar on the combs have suggested that bees store honey randomly on frames and consume nectar cells closer to the brood first when feeding the larvae [35–38]. Based on observational data on the structure of wild nests [39] and on experimental data [40] showing that bees preferred to store honey on the sides of the hive, on the inner side of the frames and on top of the brood, it has been argued that honey deposition was not completely random [41]. However, all these data were obtained from well-established hives. Monitoring how bees fill empty space during the establishment of a new colony can provide information on choices made by the colony as the brood and honey volumes expand. We show that honey is preferentially stored above the frames containing the most brood at the center of the hive, and that bees use the stores that are closer to the center of the brood first when the brood area is expanding.

## Methods

### Hives and evaluations

In November 2016, 16 hives were established in Sydney, Australia, from commercial packages containing 2kg of bees and a queen, in standard hives of type “Langstroth” containing 7 new frames with printed wax foundation comb and an in-hive feeder frame used to feed syrup to the bees during the establishment of the colonies. Colonies were fed pollen patty containing water, pollen and sugar and syrup for establishment. Colonies were fed an extra 20kg of syrup over six weeks in January and February for an experiment on neonicotinoids. These treatments had no impact on the development of the software. Frame number 7 was located closest to the feeder and consequently had a higher surface of capped honey after feeding. It is unknown whether pesticides can influence patterns of capped brood and capped honey, but no differences in development of brood and food stores were seen between treatment groups. An additional hive box was added on top in January, with no queen excluder, so that queens were left free to choose where they preferred to lay their eggs. Hives were placed on electronic scales and evaluated every four weeks to measure bee number, capped brood and capped honey area using a published method [13]. At each evaluation, frames were taken out of the hives, brushed gently to remove adult bees, placed on a rotating frame holder, and photographed with a 10Mpx Nikon D3000 camera with a Nikon 18-55mm lens and a flash Yongnuo YN-560II equipped with a light diffuser. We used three photos from a similar experiment in Tucson, Arizona, to determine whether CombCount provided similar results in different lightening conditions with a different camera. These photos were obtained with a 16.3 Mpx Pentax K-01 with no artificial light.

### CombCount implementation details

**Fig 1:**
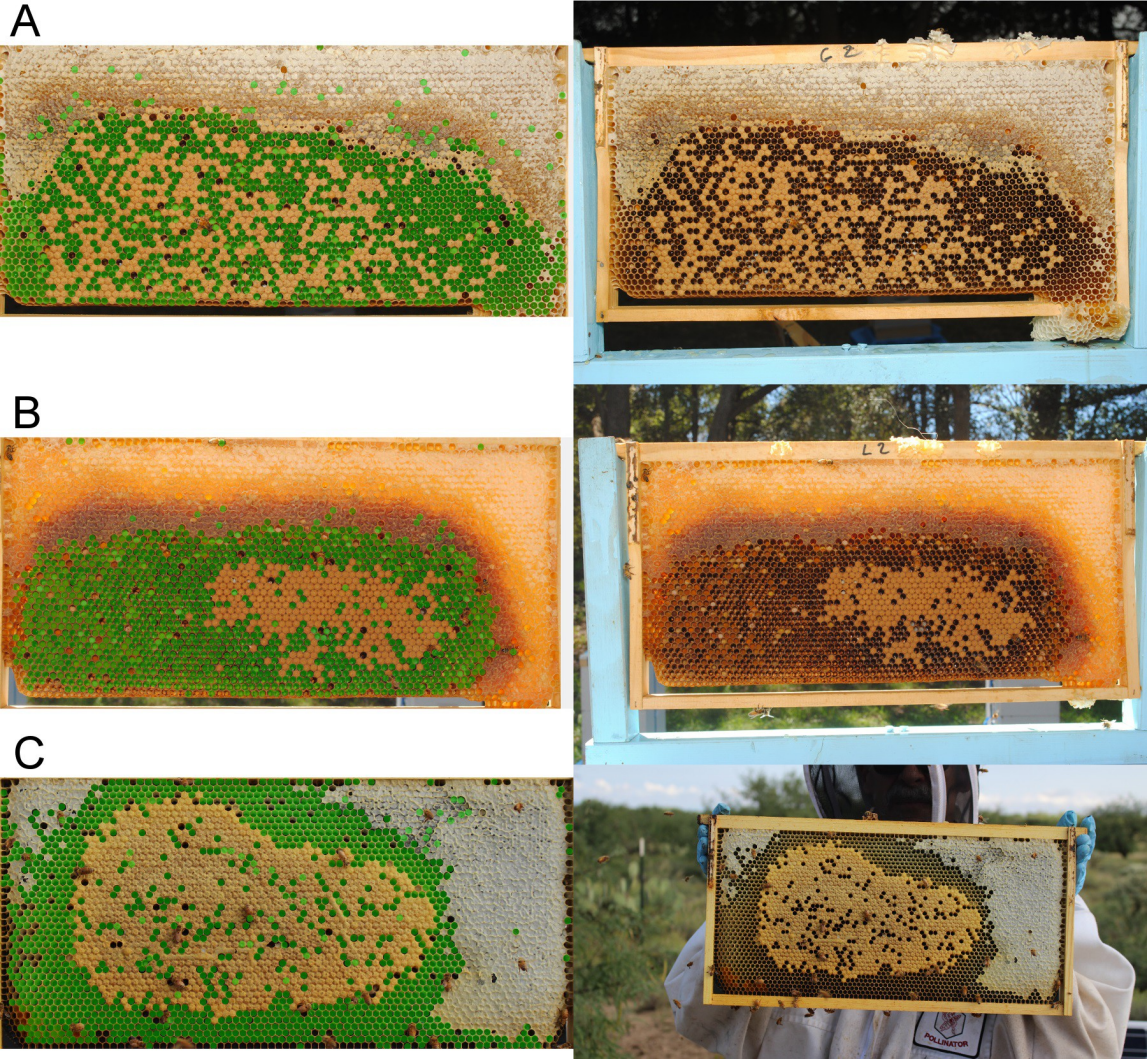
Examples of three frame photos taken in different lightening conditions and their corresponding empty cell detection estimations from CombCount. (A) Photo taken in Sydney, with the 10Mpx Nikon D3000 without flash, (B) Photo taken in the same conditions but with flash, and (C) Photo taken in Arizona, with the 16.3 Mpx Pentax K-01 without flash.

CombCount was implemented in Python with OpenCV as an image processing dependency. First, every cell of a photo of a frame was identified and labelled as either capped brood, capped honey, uncapped brood, uncapped honey, pollen or empty. Using computer vision techniques to precisely detect and classify all cells is a challenging problem, but uncapped cells exhibit particularly strong visual contrast with the surrounding texture, making them an ideal candidate class for segmentation. We found that the Hough Gradient circle detection algorithm [42] as implemented in OpenCV was reliably able to detect the boundaries of uncapped cells due to their regular and approximately circular shape, and this formed the basis of our quantification method. Using labelled images for validation, we determined that the "luminance" channel of images encoded in CIELAB (a color space designed such that Euclidean distance approximately preserves human-perceived distance between colors, where the luminance channel corresponds to human-perceived brightness) resulted in the most accurate segmentation of uncapped cells. Although this technique detects only uncapped cells, we can leverage this information to quantify the uncapped area missing from capped regions, using a small amount of human interaction to outline contiguous regions of the different classes of interest. The parameters of the Hough Gradient algorithm include an edge-detector threshold, for which we used the standard value of 200, and a circle-center accumulator threshold, which represents an adjustable sensitivity parameter. We set the circle-center accumulator threshold to an initial value of 20 and allow the human operator to adjust the sensitivity in increments of 2 to achieve the best segmentation results, which helps to account for variation in imaging conditions. The median spacing between the detected circles (median of the set of distances from each circle to its closest neighbour) was used as the radius of the circles to approximate the area occupied by the empty cells. This value is then multiplied by an inflation factor, determined by the operator, to account for variation in cell size and wall thickness between apiaries or experiments. A first phase of tests was conducted on a large sample of frame photos taken in various environmental conditions, with different cameras and lightning conditions, including with and without flash (Fig 1).

We encourage researchers to test and configure CombCount on a preliminary sample of their own photos when planning future studies. The parameter “CELL_INFLATE” allows modification of the size of the circles covering the cells, which can be useful if the size of the cells and the thickness of the walls varies across apiaries or experiments. The detection speed can be improved by changing the initial detection threshold with the parameter “THRESHOLD_INC” in case it is constantly too low or too high for a dataset. A full description of the parameters that can be adjusted is given in S1 Text.

CombCount is available online under an open-source license that allows copying, changing and redistributing the file. CombCount is accessible on Github under the name “CombCount” (https://github.com/jakebruce/CombCount) where pull requests can be submitted for improvements of the code. The original version of CombCount and instructions on how to launch it are included in S1 Text.

### Accuracy of CombCount compared to ImageJ

Thirty photos of frames were randomly selected from a dataset of 5 hive evaluations on the 16 hives established in Sydney. Photos of frames that did not contain both capped brood and capped honey were removed from the dataset and were replaced with pictures randomly selected from the same dataset (measuring empty frames is irrelevant as it is faster to enter a 0 value). If two sides of the same frame were selected, one of them was replaced, since sides of the same frames tend to be similar. Six different observers measured the 30 selected pictures with both CombCount and ImageJ. Similar amounts of training on both methods were given. Observers were given a short introductory training on how to measure the frames with ImageJ v1.51n and CombCount (with the exception of observer 1 who was already familiar with both methods) until they were confident that they could use both methods. They were asked to record the time to complete all measurements. The detouring tools are remarkably similar in both software applications and great care was given to not influence the users during the introductory training, and to give the same amount of details when explaining both methods. Half of the observers measured the frames with ImageJ first, and the other half with CombCount first. When using CombCount, users were told how to load the photos, how to select the inner corners of the frames, how to change the detection threshold until they were confident that the detection of empty cells could not be improved further, how to select honey and capped brood areas with the selection tool, and where they could access the results. When using ImageJ, users were showed how to load the photos, how to use the “rectangle” tool to measure the inner area of the frame, how to use the “freehand selections” tool to select capped brood and capped honey area, how to subtract empty areas in the middle of large honey or brood patches, and how they could save and access the results. On ImageJ, users were only allowed to use the “freehand selections” tool and did not use the “multi-point” counting tool. Although the counting tool could be more time-efficient for frames with small amounts of capped brood, the number of capped cells was often high, and the counting tool would have been slower than the outlining tool. Further, the counting tool could not have been used for capped honey cells as they were often hard to differentiate from each other. This was also done to avoid imprecision due to the conversion of individual cells into areas.

### Estimated surface of capped honey and the weight of food stores

The surface of capped honey was measured on 3328 photos corresponding to each side of each frame of 16 hives across 8 evaluations. Evaluations were conducted at least 4 weeks apart. During the two first evaluations, each of the 16 hives was made of a single box containing 7 frames and a feeder. A full depth box was added to each hive after the two first evaluations. During the last six evaluations each hive was made of the same bottom box containing 7 frames and a feeder and of a top box containing 8 frames. During the hive evaluations, each frame was gently brushed to remove the bees and weighed. We estimated the weight of the stored food alone (including pollen, uncapped honey and capped honey) on each frame by 1) subtracting the weight of the capped brood and the weight of an empty drawn frame from the weight of each frame, and 2) by multiplying the surface of capped honey measured with CombCount by the mass of honey contained in 1 cm^2^ of capped honey. Data on the weight of the brood for a given brood surface area, of the weight of an empty drawn frame, and of the weight of honey for a given honey surface were taken from published data [43]. The total mass of honey estimated with both methods was then calculated for each hive and each evaluation.

### Statistical analyses

All statistical analyses were conducted with R v 3.5.0, the figures were created using the package ggplot2 v 2.2.1.

The time each observer took to measure the 30 frames with the two different methods was compared using a paired Wilcoxon test. The area of capped brood, capped honey, and the inner frame area were compared between both methods using three separate ANOVAs, with the area of the parameter measured as a dependant variable and the method, the observers, and their interactions as independent variables. Pearson correlation tests were used to test the correlation between the measurements with the two methods for capped brood and capped honey for each observer. Because the assumption of homoscedasticity was not met, a Spearman correlation test was used to compare the surface of capped honey and the weight of food stores. The relationship between the proportion of honey stored on each frame of the top box to the proportion of brood on each corresponding frame of the bottom box was analysed with a linear mixed model, hive and evaluation were included as random effects. The proportion of the variance explained by the model was estimated [44]. The proportions of honey stored on each frame of the bottom box were compared with an ANOVA, hive and evaluation were included as random effects. Post-hoc contrasts and confidence intervals were estimated using the package emmeans v 1.2.2.

## Results

**Fig 2:**
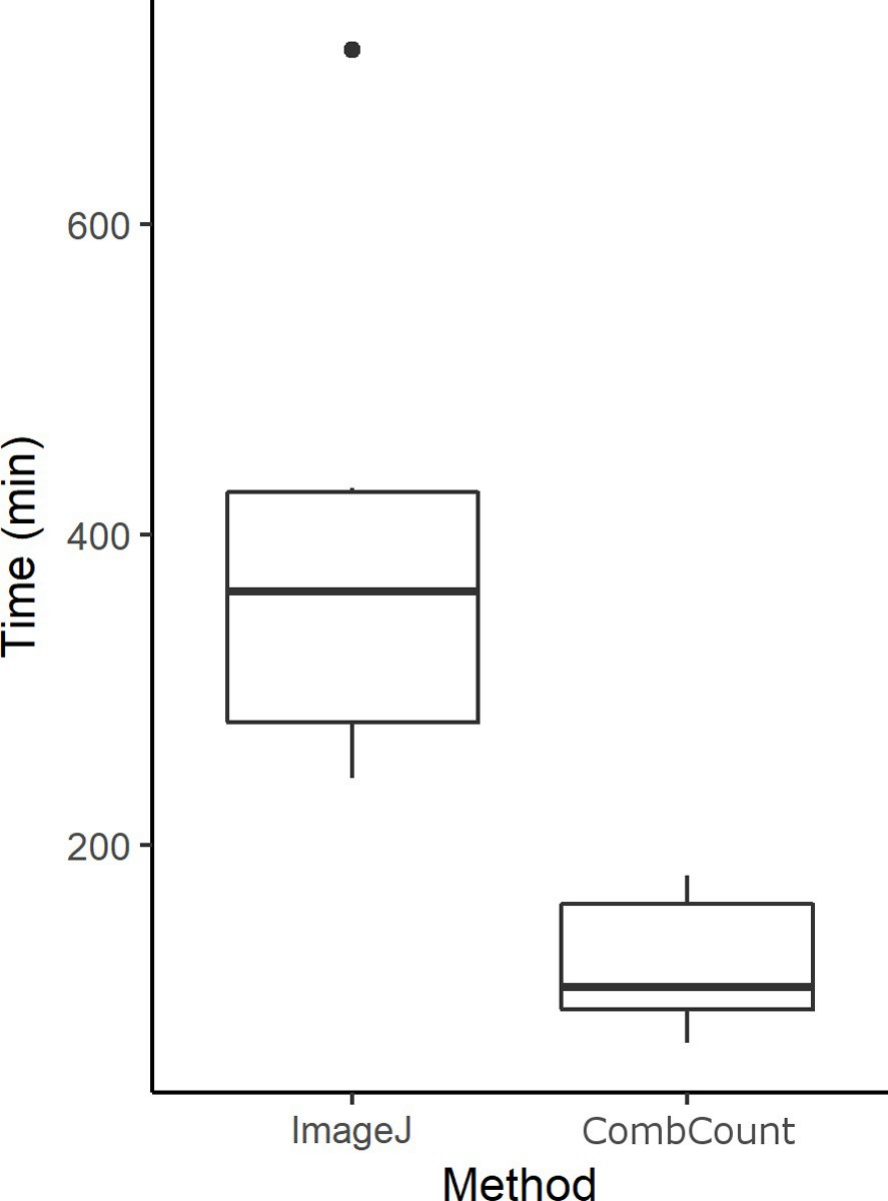
Time taken by the observers to measure 30 frames (minutes). Lower and upper edges of the boxes represent the 1^st^ and 3^rd^ quartiles respectively, the thick black lines in the boxes represent medians, whiskers extend from minimum to maximum values, one value greater than the 3^rd^ quartile by more than 1.5 times is shown as a dot.

**Fig 3:**
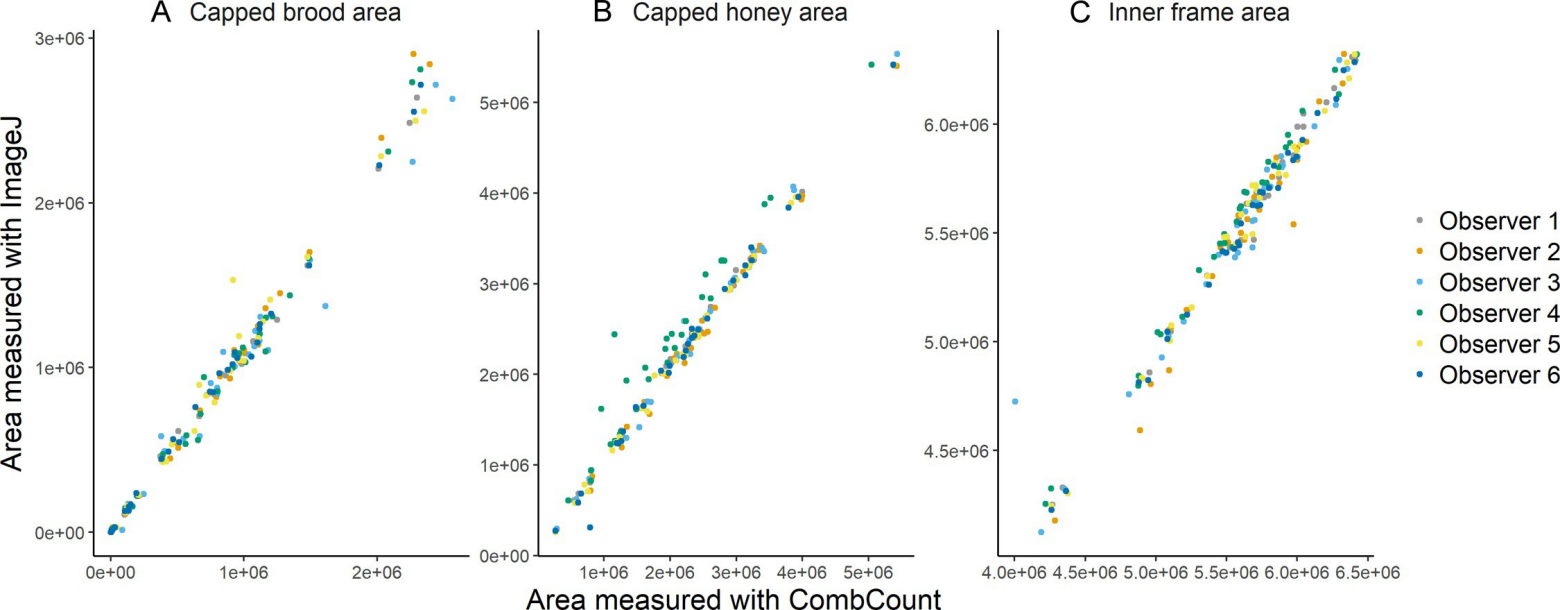
**Correlations between the measurements of (A) capped brood, (B) capped honey, and (C) inner frame area with ImageJ and Python from 6 different observers**.

Measurements were 3.2 times faster with CombCount (on average 4 min for one side of a frame) than with ImageJ (on average 13 min for one side of a frame) (Wilxocon test, V = 21, p = 0.03125, Fig 2). Measurements were correlated for all the observers, both for the capped brood area (for all observers r>0.98 and p<2.2e-16) (Fig 3A), the honey area (for all observers r>0.96 and p<2.2e-16) (Fig 3B), and the inner frame area (for all observers r>0.96 and p<2.2e-16) (Fig 3C).

**Fig 4:**
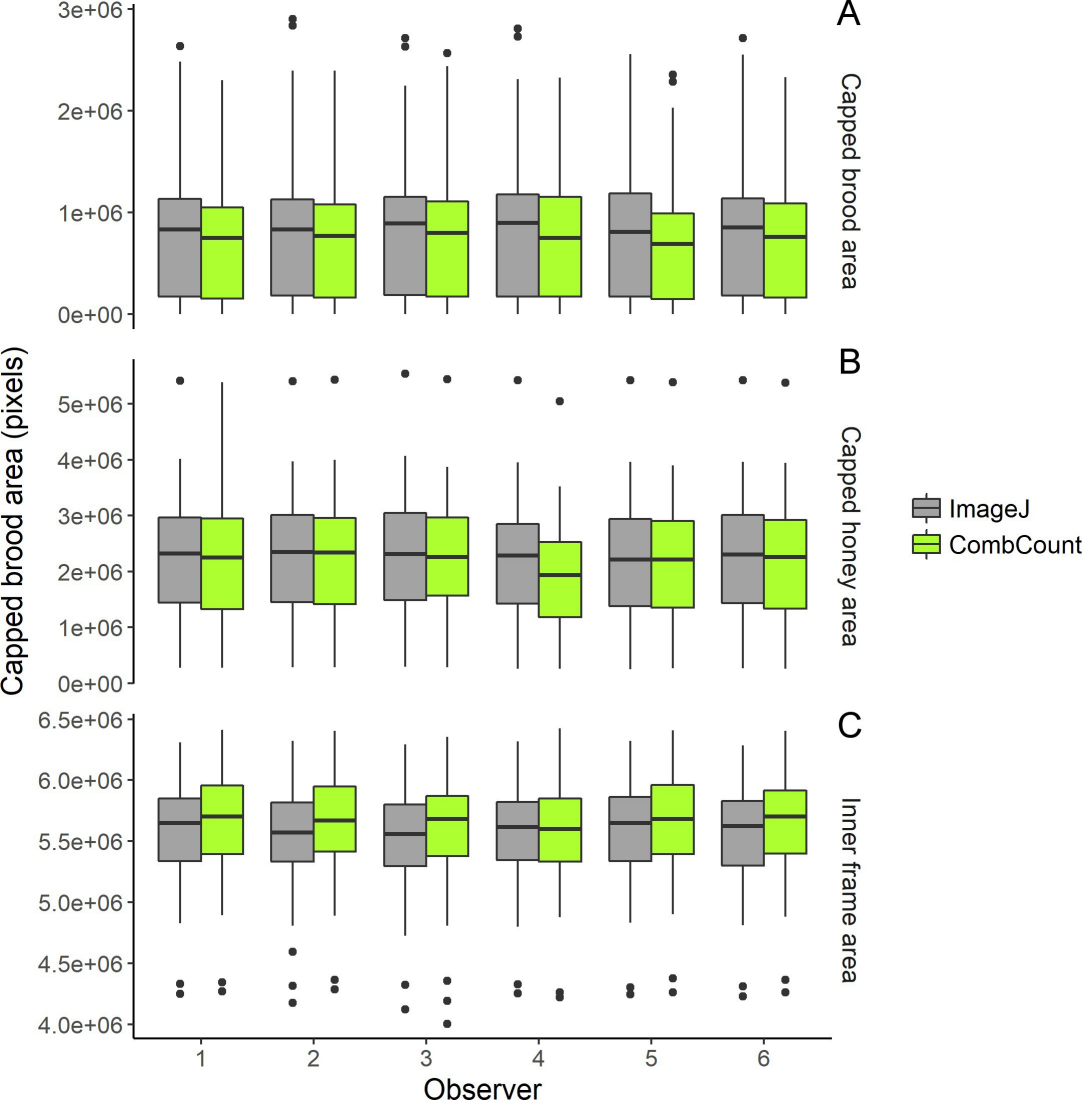
**Box and whiskers plots of (A) capped brood area, (B) capped honey area, and (C) inner frame area, with ImageJ and CombCount, by 6 different observers.** Measurements with both methods were very similar. Lower and upper edges of the boxes represent the 1^st^ and 3^rd^ quartiles respectively, black lines in the boxes represent medians, whiskers extend from minimum to maximum values, values lesser than the 1^st^ or greater than the 3^rd^ quartile by more than 1.5 times the interquartile range are shown as dots.

There was no effect of the method, the observer or of their interactions on the surface of capped brood (method: Df = 1, F = 1.309, p = 0.253; observer: Df = 5, F = 0.085, p = 0.995; interaction: Df = 5, F = 0.011, p = 1) (Fig 4A), on the surface of capped honey (method: Df = 1, F = 0.499, p = 0.481; observer: Df = 5, F = 0.33, p = 0.893; interaction: Df = 5, F = 0.196, p = 0.964) (Fig 4B), and on the inner frame area measured with the two methods (method: Df = 1, F = 1.688, p = 0.195; observer: Df = 5, F = 0.189, p = 0.967; interaction: Df = 5, F = 0.053, p = 0.998) (Fig 4C and S1 Table).

**Fig 5:**
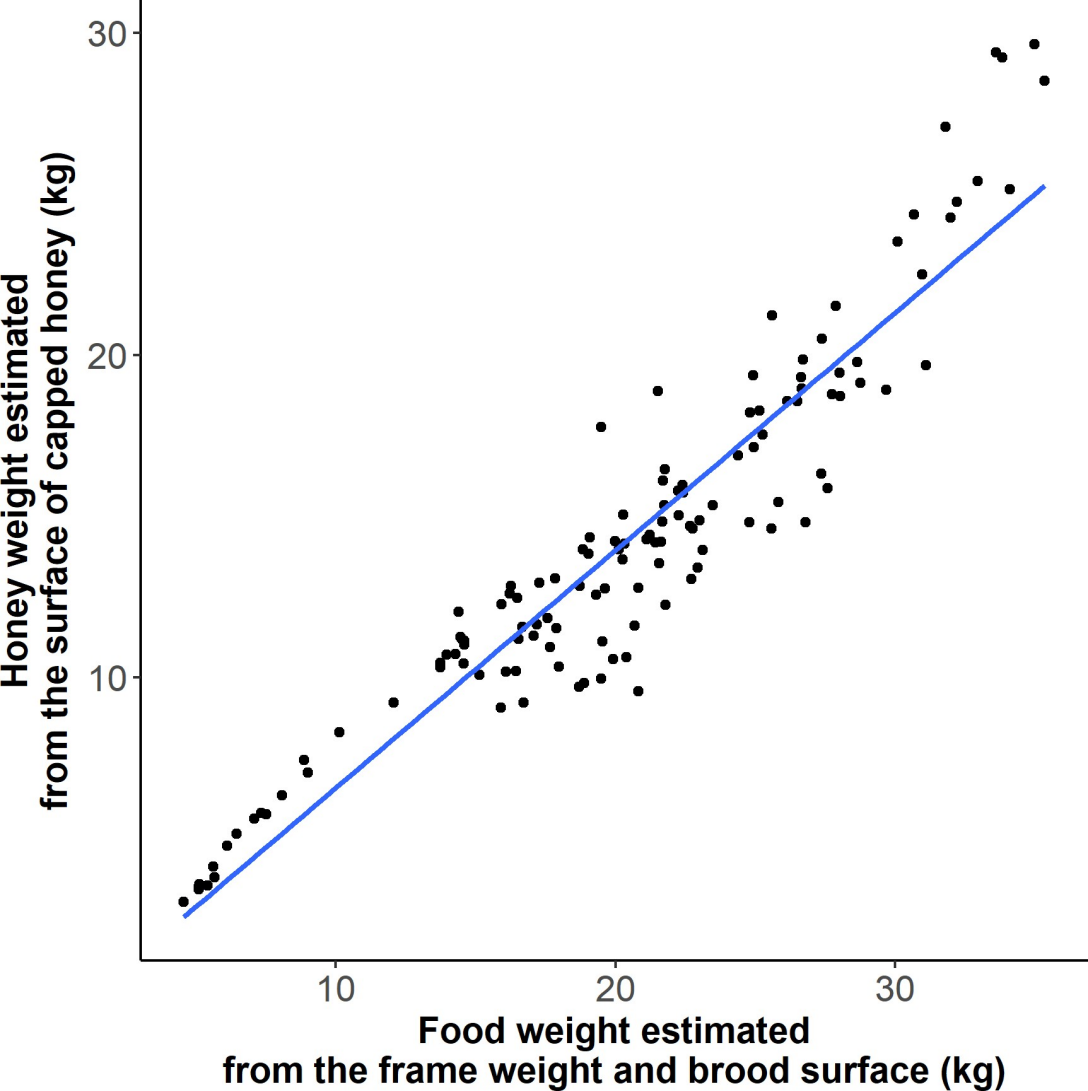
Development of a honey bee colony: Estimation of honey weight from the surface of capped honey and by subtracting the estimated weight of brood, wood, and wax from the weight of each frame measured during hive evaluations.

The surface of capped honey was correlated with the weight of food stores estimated by subtracting an estimate of weight of brood on the frame, and the known weight of the wooden and wax foundation frame materials from the measured weight of the frame (rho = 0.93, p<2e-16) (Fig 5).

**Fig 6:**
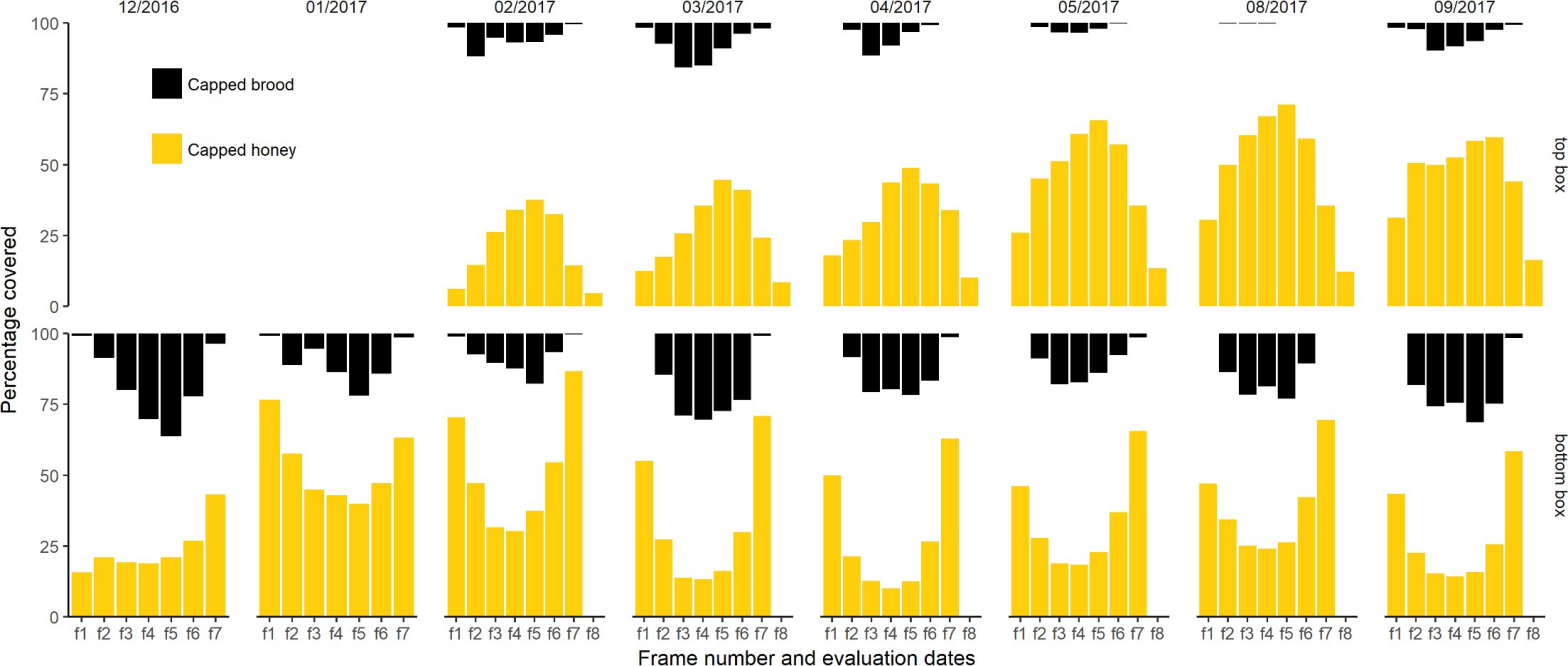
Average value of the amount of capped honey (yellow) and capped brood (grey) for each frame of the 16 hives included in this study at 8 different evaluations. A top box was added in January 2017. The 8^th^ frame of each bottom box was replaced by a frame feeder used during colony establishment and left in the hives. Hives were left undisturbed during Winter (between May 2017 and August 2017). Capped honey and capped brood are represented separately in S2 and S3 Figs.

In the bottom box, there was more capped honey stored in the frames on the sides of the hive than in the center frames, where brood was the most abundant (Figs 6 and S1 and S2 Table). There was also a significant positive relationship between the proportion of honey stored in the frames of the top box and the proportion of brood in the corresponding frames of the bottom box (estimate = 0.50, standard error = 0.05, df = 654.78, t = 9.02, p<2e-16, 2.5% confidence limit = 0.39, 97.5% confidence limit = 0.61, estimated proportion of explained variance = 0.55). Following the addition of the top box, the quantity of honey consistently decreased in the bottom box, and the area of capped brood in the bottom box decreased in the side frames (frames f1 and f7) but increased in the middle frames (frames f3 to f5).

## Discussion

The method described here, CombCount, was successfully used to measure capped brood surface area for a large, long-term colony-level study of colony development from packages in European honeybees. The results obtained were coherent with the literature on self-organized patterns on combs of honeybee colonies, and more detailed than any data previously obtained.

The patterns of capped honey and capped brood that we observed suggest that nectar storage is not random and that bees preferentially store honey in the central frames above the brood, as was previously reported [40], and that honey stores decrease on frames where brood is expanding. The first models of self-organized patterns on combs of European honey bee colonies [35,41] could not predict the patterns observed when the first generation of bees emerges or the vertical patterns of honey storage in natural hives [40,45]. Parameters such as the use of temperature by the queen to choose optimal brood cells and the difficulty of workers to keep crawling up once they reach the honey zone have been proposed to solve this issue [40,41,46]. Based on the patterns we describe, we suggest that further studies should investigate whether bees use variation in temperature to decide where to store honey. A preference for bees to unload nectar in the hottest areas could explain why we found that honey is preferentially stored on top of the brood nest and to a lesser extent on the sides, as heat diffuses and hot air rises from the brood areas to the top of the hive.

In this study we compared the speed and accuracy of measurements of capped honey and capped brood in full-size commercial bee colonies using both ImageJ and CombCount. The measurements of these two parameters with CombCount were similar to those obtained with measures using the standard ImageJ technique, across six different observers (Fig 3). CombCount decreased the time necessary to measure each frame for each observer, with measures taking 69% less time on average (Fig 2), although experienced users of the ImageJ method may observe less of a time improvement per photo. With CombCount photos can be taken quickly. They can be analysed if the light conditions allow for a contrast between the empty cells and their borders and as long as the frame is perpendicular to the lens. The ease of this method in terms of equipment needed such as flashes, cameras or tripods allows to keep the costs of photo equipment low, and to work quickly with an open commercial colony.

A commercially-available alternative to CombCount, HoneybeeComplete (WSC Regexpert, Germany) is available. HoneybeeComplete was not tested in this study and its accuracy and speed have not, as far as we are aware, been published in a refereed journal. According to guidelines provided by its authors, HoneybeeComplete requires high-quality pictures for accurate recognition of brood cells [47]. Increasing photo quality may increase camera costs, and increasing the time needed to take such photos may limit the number of colonies that can be studied at the same time. We have not here compared the utility of HoneybeeComplete and CombCount for comb measures.

The surface of capped honey was found to be correlated to the mass of uncapped and capped honey and stored pollen estimated by subtracting the weight of the brood and of an empty drawn frame to the weight of a frame. This indicates that the total food weight can be estimated from the surface of capped honey measured from photographs and confirms the accuracy of CombCount to measure the surface of capped honey.

By reducing the costs and time associated with brood and honey measurements in large apiaries, this tool will allow scientists to increase the number of research projects at the colony level, and to afford a sample size large enough to reduce the risk of false-negative results to an acceptable level.

## Supporting information

S1 Table Inner frame area, capped honey area and capped brood area measured by the different observers with the different methods on 30 pictures of hive frames. (XLSX)

S2 Table Confidence intervals and estimated marginal means for the proportion of honey stored in the frames 1 to 7 of the bottom box. (XLSX)

S3 Table Percentage of capped brood and capped honey on the frames of the sixteen hives used in this study (Fig 6), and of the honey mass stored in each hive estimated from the photos and from the frame weight (Fig 5). (XLSX)

S1 Fig Estimated marginal means (black dot) +/- standard errors (shaded area) of the proportion of honey on each frame. The degree to which arrows overlap reflects as much as possible the significance of the comparison of the two estimates.
(TIFF)

S2 Fig Box and whiskers plots of the percentage of each frame covered by capped brood for each of the eight evaluations. A top box was added in January 2017. The 8th frame of each bottom box was replaced by a frame feeder used during colony establishment and left in the hives. Hives were left undisturbed during Winter (between May 2017 and August 2017). Lower and upper edges of the box represent the 1st and 3rd quartiles respectively, black line in the boxes represent medians, whiskers extend from minimum to maximum values, numbers lesser than the 1st or greater than the 3rd quartile by more than 1.5 times the interquartile range are shown as dots.
(TIFF)

S3 Fig Box and whiskers plots of the percentage of each frame covered by capped honey for each of the eight evaluations. A top box was added in January 2017. The 8th frame of each bottom box was replaced by a frame feeder used during colony establishment and left in the hives. Hives were left undisturbed during Winter (between May 2017 and August 2017). Lower and upper edges of the box represent the 1st and 3rd quartiles respectively, black line in the boxes represent medians, whiskers extend from minimum to maximum values, numbers lesser than the 1st or greater than the 3rd quartile by more than 1.5 times the interquartile range are shown as dots.
(TIFF)

S1 Software Program file written in Python containing the software CombCount. (PY)

S1 Text Instructions to launch CombCount and change the default parameters of the software. (DOCX)
