# Supplementary figures and images for: The development of honey bee colonies assessed using a new semi-automated brood counting method: CombCount
Source: PLoS One. 2018 Oct 16;13(10):e0205816. doi: 10.1371/journal.pone.0205816 (PMC6191133; doi:10.1371/journal.pone.0205816)

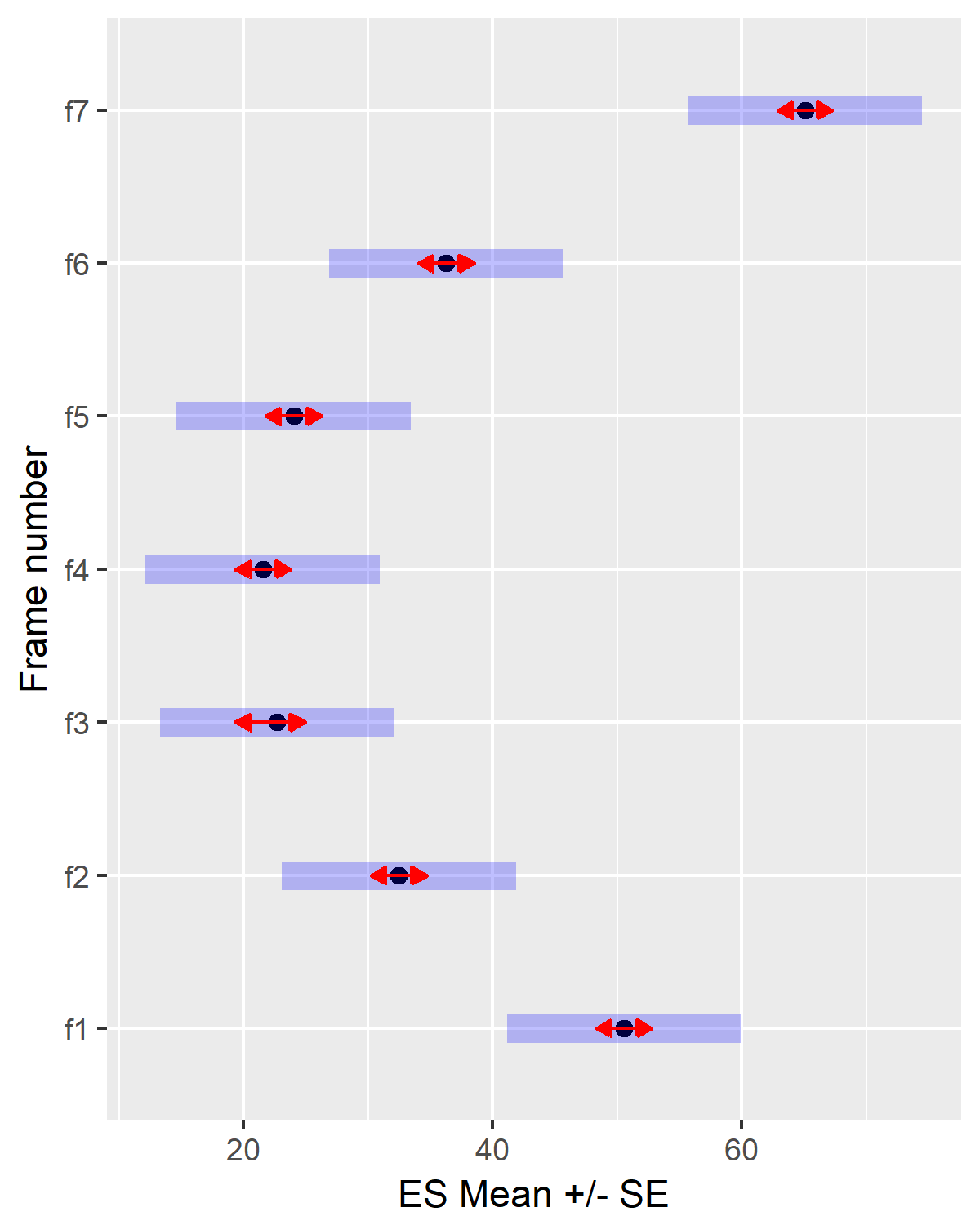

Supplement: S1 Fig — The degree to which arrows overlap reflects as much as possible the significance of the comparison of the two estimates. (TIFF) [file pone.0205816.s002.tiff]

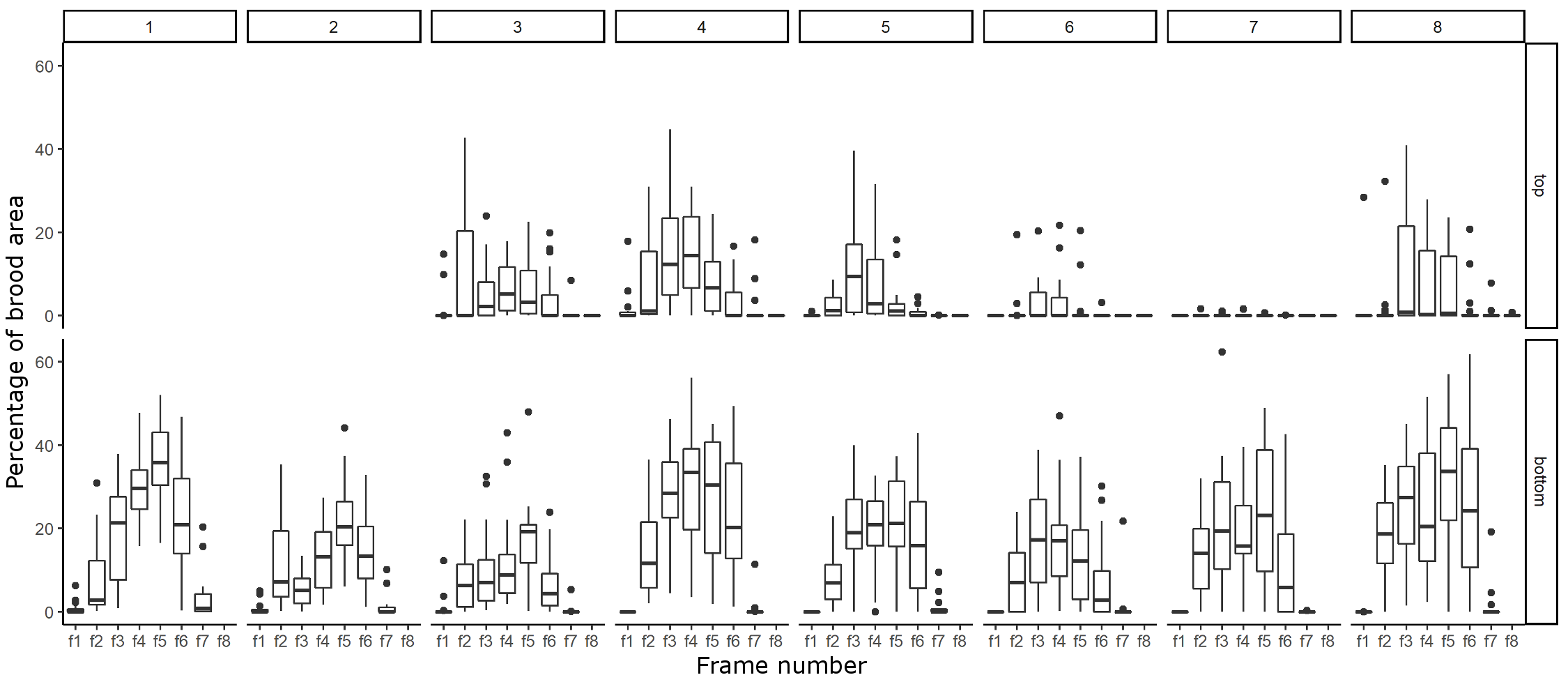

Supplement: S2 Fig — A top box was added in January 2017. The 8th frame of each bottom box was replaced by a frame feeder used during colony establishment and left in the hives. Hives were left undisturbed during Winter (between May 2017 and August 2017). Lower and upper edges of the box represent the 1st and 3rd quartiles respectively, black line in the boxes represent medians, whiskers extend from minimum to maximum values, numbers lesser than the 1st or greater than the 3rd quartile by more than 1.5 times the interquartile range are shown as dots. (TIFF) [file pone.0205816.s004.tiff]

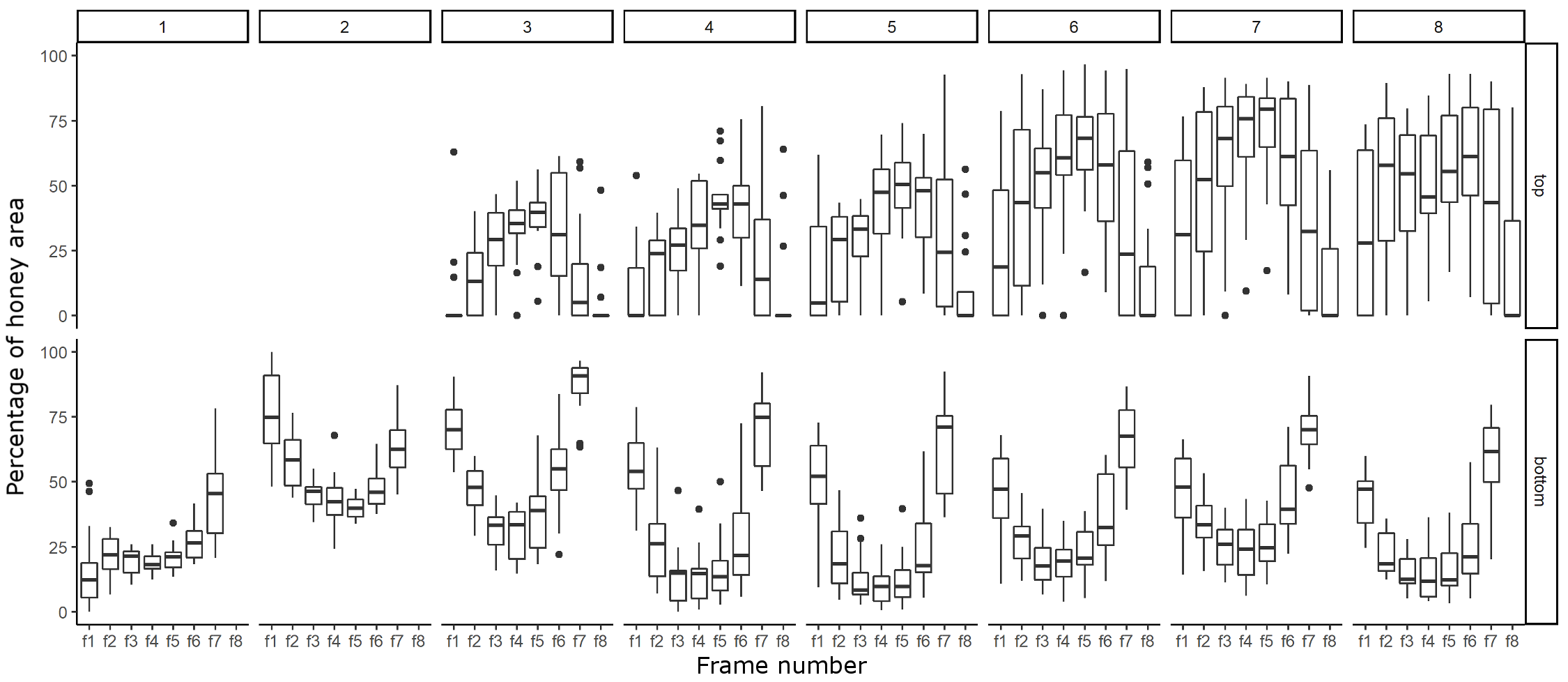

Supplement: S3 Fig — A top box was added in January 2017. The 8th frame of each bottom box was replaced by a frame feeder used during colony establishment and left in the hives. Hives were left undisturbed during Winter (between May 2017 and August 2017). Lower and upper edges of the box represent the 1st and 3rd quartiles respectively, black line in the boxes represent medians, whiskers extend from minimum to maximum values, numbers lesser than the 1st or greater than the 3rd quartile by more than 1.5 times the interquartile range are shown as dots. (TIFF) [file pone.0205816.s005.tiff]
