# Supplementary material for: The development of honey bee colonies assessed using a new semi-automated brood counting method: CombCount
Source: PLoS One. 2018 Oct 16;13(10):e0205816. doi: 10.1371/journal.pone.0205816 (PMC6191133; doi:10.1371/journal.pone.0205816)
Supplement: S1 Text — (DOCX) [file pone.0205816.s007.docx]

**Launching CombCount**

Python 2 or 3 and the Numpy and OpenCV dependencies need to be previously installed. On a Windows platform, this can be done easily by installing Python distributions that include these dependencies, such as Python (x,y) (https://python-xy.github.io/).

CombCount must be launched with Python in a terminal and for a single picture. After opening the terminal, the observer must indicate the working directory and then call CombCount with:

>python S4_Software_CombCount.py your_image_name.jpg

The frame and side numbers (in this order) can be added after the name of the image when launching CombCount, they will be returned with the output:

>python S4_Software_CombCount.py your_image_name.jpg frame_number1 side_1

After launching CombCount, the observer is invited to manually select the four inner corners of the frame. CombCount overlays a green semi-transparent circle of the size of a cell on every cell that CombCount recognises as uncapped (Fig 1). The observer can then decrease the circle-center threshold value if more cells should be recognised as uncapped, or increase this value if capped cells have been overlayed with a green circle. Once the observer is satisfied with the result, usually when there are no more false-negative and false-positive detections, or when the number of false-positive equals the number of false-negatives, two polygons must be drawn, one around the honey and one around the brood. The surface of the green circles is automatically subtracted from these two polygons and the capped honey and capped brood areas are returned along with the inner frame area in the terminal. Two images, representing the area of capped brood and of capped honey, are saved in the working directory. A third file containing the resolution of the image, the points that describe the brood and honey polygons, the detected circles (location and radius) that represent the detected empty cells, and the median between them, is saved in the same folder as a standard Python Pickle file.

**Parameters of the software**

Some parameters of CombCount can be edited at the beginning of the “S4_Software_CombCount.py” file:

INITIAL_THRESHOLD: circle-center accumulator threshold, this threshold can be increased or decreased by the observer to improve the detection of empty cells. This value should be changed if there are constantly too many false positive or false negative cell detections on the photos of a same series.

THRESHOLD_INC: amount by which the threshold is increased or decreased by the observer.

DS: downsampling factor, speeds up the computing time required to detect the circles, but might decrease the accuracy of the results.

PADDING: padding of the instruction messages.

POLY_ALPHA: opacity of the selected polygons.

CIRC_ALPHA: opacity of the detected circles.

CELL_INFLATE: cell size inflation value.

DETECT_DS: downsampling factor for the circle detection, speeds up the computing time required when adjusting the circle-center accumulator threshold, but might decrease the accuracy of the results.

REDETECT_ON_FULL_IMG: activates or inactivates the redetection of the circles on the full image (only useful if a downsampling has been used).

FULLSCREEN: activates or inactivates the fullscreen mode.
